# Medico-Legal Trial—Plea, "Lunacy"

**Published:** 1858-10-01

**Authors:** W. J. J. Leach


### MEDICO-LEGAL TRIAL—PLEA, "LUNACY."

### CASE OF THE REV. W. J. J. LEACH.

[It was our intention to have entered fully into the consideration of the case of Mr. Leach, but as a verdict of sanity has been recorded by the jury, we will give this gentleman all the benefit of this decision, and say nothing that can further affect his social position. We append a report of the evidence as it appeared in the public journals at the time.]

A commission was opened on Thursday before Mr. Barlow, one of the Commissioners in Lunacy, at the Gray's Inn Coffee-house, May 20th, 1858, to inquire whether the Rev. William James John Leach was of sound mind and competent to dispose of his property. The commission was issued upon the petition of Julia Caroline Leach, the mother of the supposed lunatic, and at whose death he will come into possession of nearly 50,000*l.* in the funds.

### EVIDENCE IN SUPPORT OF THE LUNACY.

Dr. Forbes Winslow said—I was first consulted in reference to Mr. Leach in May, 1853. He resided at that time at Upper Southwick Street, Hyde Park.

He was at that time in a state of mental aberration, and an attendant had the charge of him. His mind appeared to be morbidly excited upon religious subjects, and I have no doubt that he was insane. He was, in my opinion, labouring under several delusions connected with religious questions. I saw him again in 1856, when he then was living in a cottage at Hammersmith by himself, and I had some long conversations with him, which satisfied me that his mind was in the same state as in 1853. He said that the millennium was at hand, and that our Saviour would soon be upon earth; that all social ranks and distinctions of society were abolished; and he called my attention to a paragraph in the *Record* newspaper, which stated that an old woman had seen our Saviour near Bridgewater. He appeared to believe that this statement was true. At this time, Mr. Leach having exhibited no marked excitement or disposition to acts of mischief to himself and others, I advised that no step should be taken to restrain his liberty, but I directed that he should be carefully watched, in the event of his committing some overt act of insanity. A few weeks after I had given this report and advice, I was again consulted by the family, who represented they had ascertained that Mr. Leach had made an offer of marriage, and had actually engaged himself, to his housemaid! Recognising Mr. Leach to be in an insane state of mind, and quite incompetent, in consequence of his insanity, to act with a sound judgment in such matters, I suggested that he should be placed under supervision and control, but advised, before doing so, that two independent medical men should be called in to examine him, and certify to his condition before he was removed. He was subsequently placed in my private asylum. While he was in my establishment I had frequent conversations with him. He did not appear to think that the millennium had actually arrived, but that it was dawning, and he said that there was so much wickedness and fraud in this world at the present time, that it clearly showed the millennium must soon arrive. He said he did not actually believe that the old woman had seen our Saviour, but he said he should like to go and inquire into the subject. He then told me that many years before he had seen a vision of our Saviour on the cross, as God manifest in the flesh, while in the pulpit, and that the vision had converted him. I spoke to him upon the subject of his wearing his beard, and asked him to cut it off; and he said that while translating the Scriptures some years before, an accident had occurred, the nature of which he would not explain, and that a revelation was then made to him respecting his beard, and he now continued to wear his beard in obedience to Divine command, and in deference to the sanction of the Holy Ghost and Spirit; that on one occasion he had cut off his beard, and that he had suffered in consequence the greatest mental agony; and he considered he should be committing a great abomination to the Almighty if he were to do it again. He was also under the impression that there were five distinct voices within him which directed all his actions, and that one of them in particular was a special one, which directed him in every act of his life, whether trivial or important. I endeavoured to analyse his ideas, and to ascertain whether he alluded to the voice of conscience or the voice of reason or judgment; but he said the voices he referred to were nothing of this kind, but were special to himself. He said none of the voices were audible ones. He also told me that it was his custom to pray in an erect position, for several hours, in the middle of the room, with his arms in front of his head, but he only prayed mentally, and did not utter a word; and when he was tired of standing, he prostrated himself on his face. He said that he generally prayed for the restoration of the miraculous gifts to the Church, and he expected that the result of his earnest prayers would be that he as part of the Church would have the power to bring the dead to life, to restore sight to the blind, and to heal the sick. He spoke about his servants, and said they ought to be treated more kindly than they were, and more as equals, and that he dined and took his meals with his servants and kissed them in the morning, and allowed them to sit on his knee. He also said that after family prayers he had his servants in the drawing-room and played cards with them until three o'clock in the morning, and between the deals he read chapters out of the Bible to them. I told him that such proceedings as those were contrary to the views entertained by gentlemen and persons in his position, and that they were not consistent with the position of a gentleman and a clergyman; and he replied, that it was part of his religious course of life so to comport himself with his servants. He also said that he was engaged to be married to one of his maids, and he said that he kissed this one upon the lips and the other upon the cheek. I then asked him whether, supposing he should be set at liberty, he would purchase pistols again, and he said he certainly should, and that he should carry his gunpowder in his waistcoat pocket in order that it might be kept dry. His mind appeared so much disordered by religious impressions, that it was very difficult to get him to converse upon any other subject. He displayed a good deal of shrewdness, which was very common with lunatics, who very frequently exhibited great caution and cunning in concealing their hallucinations. Dr. Winslow, in conclusion, said he had no doubt that Mr. Leach was of unsound mind, and that he was quite incompetent to manage his affairs or to take care of his property.

Dr. Winslow was subjected to a long cross-examination by Mr. Chambers. He said he had no doubt that Mr. Leach would be able to solve any proposition of "Euclid" that might be placed before him; but this, he said, would not at all alter his opinion of the state of his mind. He advised his family not to put him under restraint until he committed some overt act of insanity, and the overt act he committed was promising to marry, and being about to marry, one of his servant maids. Witness considered that the servants had exercised undue influence over a man whose mind was affected, and that he had been entrapped into making the promise to marry, and he thought it his duty to interfere to prevent a man in an insane state of mind from committing himself in such a manner. He advised that two independent medical men should be called in to examine Mr. Leach, and give the necessary certificate to enable his friends to place him under restraint. He was not aware that one of the gentlemen who signed the certificate had been the assistant and was now the partner of Mr. Sidden, the brother-in-law of Mr. Leach, but he believed it had been so stated. He should not think of reasoning with a man who told him that the millennium had arrived, because he did not think that any man who came to such a conclusion, contrary to the evidence of his own senses, was a fit subject to be reasoned with. He was aware that many eminent men, divines and others, had expressed very extraordinary opinions upon the subject of religion, and he did not form his conclusions as to the insanity of Mr. Leach from any one particular fact, but from all the circumstances connected with the case. If any man, however, were to tell him that he had seen our Saviour upon the earth, he should consider it a very grave circumstance in reference to the state of his mind. The vision he referred to he represented he had seen twenty-seven years before. Witness had some conversation with him in reference to his wearing a beard, and he said that it was effeminate to cut off the beard, and he quoted several passages from the Scriptures referring to men making themselves like women, in confirmation of what he stated. He afterwards said that, while engaged in translating the Scriptures, he found a particular word which justified him in supposing that it was the Divine command that he should wear his beard, and that he did so by the Divine authority. Mr. Leach also had the delusion that he had been mainly instrumental in promoting the abolition of the punishment of death for all crimes, and he considered that he had effected this by writing a letter to the *Times*, which was never inserted, and by conversations he had had with persons in omnibuses upon the subject. With regard to his intended marriage, he said that he had promised one of his servants to marry her, and that it would be disgraceful and dishonourable not to do so. He also said that he had seen the father and mother of his intended bride, and that they had consented to the marriage, and everything was arranged, when his mother interfered and got him shut up in a madhouse. Witness told him that he could not expect any happiness from such a union, and he replied that the girl was a very well conducted young woman, and he believed he should be very happy with her. Dr. Winslow then said he did not mean to have it understood that he considered it by any means an indication of insanity that a man of fifty-four should marry his servant; but he coupled the fact with the history of the case and with Mr. Leach's delusions on several topics, and this led him to the conclusion that Mr. Leach was not of sound mind, and unfit to manage himself and his affairs. He said that Mr. Leach appeared to be quite aware that he would come into possession of a large sum of money at the death of his mother, and he appeared to desire to sell the reversionary interest for an annuity for his life. He appeared to have a great horror of his mother, and he understood that they had quarrelled on account of her refusing to allow the servants to take their meals with him. Mr. Leach might have been able to pay his bills, and conduct operations of that description for twenty or thirty years; but this fact would not at all affect his opinion with regard to the state of his mind. When Mr. Leach talked about praying, he did not allude to the present inquiry, and he did not remember hearing him say that he had prayed to God earnestly, for an hour or more, that the inquiry might end in his being released. At the time Mr. Leach procured the pistols there had been a good many robberies committed at Hammersmith, and his own house was broken into on one occasion; and he said he had procured the pistols for his own protection. He believed Mr. Leach possessed extraordinary mental powers and literary attainments, and that he devoted himself a great deal to study.

Mr. Henry Sidden, a medical gentleman, the brother-in-law of Mr. Leach, was then examined. He deposed that he had married Mr. Leach's sister, and he had known him for twenty years. He then proved that in 1841 he was attacked with madness, and locked himself in his study, and when the door was opened he was found quite naked. He was under restraint at this time for a short period, when he recovered. He was again attacked in a similar manner in 1852, and a third time in 1853, and he broke all the windows in his room, and he said he did this in order that the neighbours might hear him play the flute. He had an enema syringe in the room which he represented to be a flute, and he broke it in endeavouring to play upon it. When he was attacked in 1853, Dr. Winslow was called in, and under his advice an attendant was provided for him, and he was closely watched. The witness also proved that Mr. Leach was continually talking about the millennium and other religious subjects, and also about an old woman having seen our Saviour at Bridgewater, and he appeared to believe that she had done so. His mother was seventy-three or seventy-four years old. In 1856, Dr. Winslow was again consulted, and upon his reporting him to be insane, witness took the necessary steps to obtain a certificate to authorize his removal to a place of confinement. The certificate was signed by Mr. Gray and Dr. Wood. The former was at that time house surgeon at Guy's Hospital, and the latter had since been appointed physician to Bethlehem Hospital. Mr. Gray had since become witness's partner.

Cross-examined—In the event of Mr. Leach dying unmarried, and without a will, a great portion of his property would come to witness's wife. In June, 1856, Mr. Leach executed a deed, under which he received a sum of 1194*l.*, and witness received a similar amount. Upon the death of Mr. Leach's mother, witness's wife would be entitled to 17,000*l.*, and Mr. Leach would receive 30,000*l.*, and if he should die unmarried, and without a will, witness believed that sum would go to his wife. He and his wife went to visit Mr. Leach while he was at Dr. Winslow's establishment, and he was very violent, and his wife was very much frightened. He was violent on account of his having been placed in a lunatic asylum; but witness did not recollect that he said that he and his family had shut him up there to prevent him from marrying his servant-maid. He had been doing duty at one of the churches in the neighbourhood down to the period of his first attack, and he ascribed this attack to the excess of labour that devolved upon him owing to the illness of the rector, and other causes. He first heard of Mr. Leach's intention to marry one of his servant-maids in January, 1857, and it was after this that Mr. Gray and Dr. Wood signed the certificate of his insanity. He knew perfectly well what he was about when he executed the deed under which he obtained the 1200*l.*, and was not under any delusion. Witness invested the money for him with the exception of 100*l.*, which he handed to him. Dr. Winslow advised him to get the certificate of insanity signed by two independent medical men, and he considered Mr. Gray was perfectly independent, although he had been his assistant. Mr. Leach, on every occasion when he was free from the surveillance of the keepers, complained of being put in such a place of confinement, and said that he was no more mad than witness was. He desired to dispose of his reversion for an annuity of 800*l.* a year, and if he had done so, of course it would have prevented his wife from having a chance of getting the money.

In re-examination the witness said that his wife was in very delicate health, and Mr. Leach was much more likely to live than she was. She was at present too ill to attend to give her evidence.

Some other evidence was then adduced, showing the nature of the attacks in 1841, 1852, and 1853, and it appeared that, on the two former occasions, it was found necessary to put a strait-waistcoat on him. On the third occasion it appeared that the attack was not of so severe a character.

Elizabeth Burn, a person previously in the service of the alleged lunatic, proved that on several occasions she and the other servants played at whist with him and that during the intervals of the games he read chapters from the Bible. She also stated that he said there was no harm in playing cards if people read the Bible at the same time.

Dr. H. Southey, one of the Lord Chancellor's Commissioners in Lunacy, was then examined.—He confirmed the evidence given by Dr. Winslow as to Mr. Leach's delusions.

Dr. Wood, formerly the medical officer of Bedlam, deposed that he had several interviews with Mr. Leach with a view to ascertain the state of his mind. In the course of the conversations that took place between them, Mr. Leach said that everything he did was under the influence of the Holy Spirit, and that this Spirit controlled every action of his life. The pistols, he said, were purchased by him for self-defence, and he kept two of them upon the chimney-piece in the dining-room as a sort of chimney ornaments. In the course of the conversation he said that he should be justified in shooting any man who trespassed upon his field after evening; and he also said that he prayed that any man who might come into his house in the dead of the night might be brought to the muzzle of his pistol that he might shoot him dead. Witness remarked that it might occur that a man was in the house or the field innocently, and he said that did not signify, for he said he was sure no man could be killed who was innocent; and that, if they were not guilty of any offence at the time, they had committed an offence at some other period for which they deserved to die. He made the same observation with regard to persons who were executed. He accounted for wearing his beard by stating that God was displeased with him for his effeminacy in cutting off his beard, and said that the Scriptures commanded that men should not assume the garb of the other sex. Mr. Leach also said that the reason he had the servants to take their meals with him was to humiliate his mother, who had insulted him the day before. During the conversation witnessed asked him if, supposing the present matrimonial arrangement was put an end to, and he were to desire to introduce a nice young lady to him, whether he would consent to the introduction without consulting the Holy Spirit, and he replied that he certainly should not, and if the answer were affirmative, he should at once consent to see the young lady; but if it was negative, he should say, "No, I thank you; I would rather wait a little time." He also said that it was at the instigation of the Holy Spirit that he took his servants upon his knees and kissed them. Dr. Wood concluded by stating that there was every characteristic of insanity about Mr. Leach, and the impression upon his mind was that he was a very dangerous lunatic.

Cross-examined—Witness did not state at the last inquiry that he considered Mr. Leach a dangerous lunatic. He was always of opinion that he was of unsound mind, but what had recently come to his knowledge strongly confirmed his original opinion, and also induced him to form the conclusion that he was a dangerous lunatic. He saw him on the 17th and 18th of the present month. The first interview occupied three hours, and the second more than two hours. He first saw Mr. Leach in January, 1857. He did not tell him what his object was. Lunatics possess so much cunning that, if he had been made aware of his object in visiting him, Mr. Leach would in all probability have corrected the hallucination under which he was labouring. He introduced himself by stating that he had understood that Mr. Leach possessed some peculiar opinions on religious subjects, and he wished to converse with him upon the subject of the millennium, and he appeared to have his mind so full of that subject that he readily entered into conversation respecting it. During the conversations they had together, Mr. Leach repeatedly expressed himself very angry with his mother for treating him as an insane person, and he said that she had been very cruel and unjust towards him, and that she was actuated by interested motives, and was improperly influenced by other persons. He also said that the young woman to whom he was engaged to be married was a very respectable young woman, and he considered that he was morally bound to marry her, and that it would be dishonourable in him to break the promise he had made to her. He would not swear that Mr. Leach did not say that he knew that a man could not shoot another for committing a trespass, and that a man had been hanged for shooting another who had trespassed upon his field.

Re-examined—The ground upon which he formed the opinion that Mr. Leach was a dangerous lunatic was, that he possessed himself of pistols, and said that no man could be destroyed who was innocent; for if he was innocent of the particular offence for which he was destroyed, he had no doubt committed some other offence for which he deserved death.

Dr. A. J. Sutherland, Physician to St. Luke's Hospital, was examined by Mr. Booth, Q.C.

I visited Mr. Leach December 8th and 9th, 1857, and on the 15th instant, for the purpose of examining the state of his mind.

December 8.—Mr. Leach said that in order to humble Mrs. Leach, and to show that he was master in his own house, he made the servants breakfast with him, and induced his hostler to play the Hallelujah Chorus to them one evening, and played a rubber of whist with the servants and a dressmaker. I asked whether this had any reference to the millennium; he said that servants will be differently treated during the millennium, that after opening the door at the close of the day they will be permitted to associate with the family. He said that he had prayed frequently and fervently that the miraculous gifts of the Holy Spirit might be restored to the Church; that if the old woman who was stated to have seen Christ in the neighbourhood of Frome were a credible witness, he would believe that she had seen Him. He said there can be no mission without miracles; raise my wife from the dead, and I will believe in the truth of a prophet's mission.

He said that he had two brace of pistols, a pocket-pistol, and a revolver; that the latter he had not seen for some time; that he was in the habit of carrying his powder-flask in his fob; he said, "Put your trust in Providence, and keep your powder dry." He said that after long prayer he had received a deep intimation or sanction to allow his beard to grow, that about two years ago he had doubts upon the subject, and shaved it off, and that for several nights in succession he awoke, and experienced great terror of mind, as he thought that he had not acted up to the intimation and sanction he thought he had received.

December 9.—He told me that the intimation and sanction was as distinct as if he heard an internal voice—the still small voice of the Spirit—that it was an internal impression, that if he waited expectantly and with patience, he received the sanction for everything he did—*e.g.*, in ordering dinner. How far this power would be increased when the Almighty gave further evidence of the millennium, he said he did not know.

I saw Mr. Leach soon after the first inquiry at the Holland Arms, when he said that I had mistaken what he had said respecting the voice. I said I did not know how that could be as I had written down what he said in his presence, and had read what I had written over to him. He said there are four kinds of voices—The ordinary voice when we are speaking; the voice when we speak to ourselves; the voice when we think, and this voice.

May 15.—Mr. Leach said that the present was the dawn of the millennium. I asked what were his proofs. He said his own experience, that formerly when he prayed he prayed like any other man, but that now he feels a perceptible influence, the words he uses are by inspiration; and that if I were to kneel down to pray, that I should do so without any supernatural influence. He said that he was under inspiration when he translated the Bible; that there was a mistake, which induced him to pause; that it created a shock because it raised a doubt in his mind as to the supernatural influence, but that now he has no doubt that he was under the influence of the Spirit when he made the translation of the Bible. He again alluded to his having shaved off his beard. He said that a supernatural terror arose in his mind for two or three nights in succession, so that he had experienced a supernatural terror and a supernatural joy; the joy was when he saw the Saviour on the cross about thirty years ago.

I asked what other proof he had of inspiration? He answered that his hand was directed as well as his voice in the translation of the Bible. He said that his voice in prayer is influenced by the Holy Spirit. The same influence which induces him to pray as he does now, induces him to allow his beard to grow, and did induce him to translate the Bible.

Mr. Bovill.—What is your opinion of the state of Mr. Leach's mind?

Dr. Sutherland.—I consider that he is of unsound mind.

Mr. Bovill.—Do you think him capable or incapable of managing himself and his affairs?

Dr. Sutherland.—I consider him incapable of managing himself and his affairs.

Mr. Bovill.—Explain why you think Mr. Leach is incapable of managing his affairs.

Dr. Sutherland.—We are obliged to regard the bearings of the delusions upon the conduct of the patient, and although I agree with Mr. M. Chambers in thinking that the knowledge of the amount of a person's property, and the payment of his bills is an element in such consideration, yet there have been instances where persons possessing such knowledge have been found of unsound mind by juries, and incapable of managing themselves and their affairs.

No accounts could have been better kept than those of Mr. Devonport, and Mr. Gundry knew to a fraction the amount of his property. I repeat what I said on a previous occasion, that the delusions under which Mr. Leach labours render him peculiarly liable to become the prey of designing persons, and may render him dangerous to others.

Cross-examined by Mr. M. Chambers.

Dr. Sutherland.—I do not consider the particular doctrines of the Irvingites, Quakers, and Swedenborgians to have any bearing upon the case. Mr. Leach is a clergyman of the Church of England, and must be judged by his antecedents.

Mr. Chambers.—But supposing Mr. Leach did not consider it right to keep Sunday in the usual way, would not that have a bearing upon the case?

Dr. Sutherland.—It would have this bearing upon the case, that if Mr. Leach holds the doctrines to which you refer, he would not be allowed to do duty as a clergyman of the Church of England.

Mr. M. Chambers.—You say that Mr. Leach is likely to become the prey of designing persons; how does Mr. Leach's mind differ from that of any other person?

Dr. Sutherland.—We are guided by the ordinary operations of the Holy Spirit. Mr. Leach thinks that he is guided by the extraordinary operation. Our judgment is assisted, Mr. Leach considers that his judgment is superseded by the Holy Spirit.

Mr. M. Chambers.—Did you write down at the time that Mr. Leach thought himself inspired?

Dr. Sutherland.—I did.

Mr. Bartlett, the medical officer of Dr. Winslow's asylum, proved that he took charge of Mr. Leach in January, 1857, and he remained under his care until December of the same year. During that period the Commissioners of Lunacy had four or five interviews with Mr. Leach, and he had ample opportunities of communicating with them. He was eventually removed to a private lodging, under the authority of the commissioners, in the charge of an attendant, and under Dr. Winslow's superintendence. When he went to remove Mr. Leach in the first instance, he said that if he had known the object of his visit he would have shot one or two of them. Mr. Leach afterwards denied having made this statement. This witness also spoke to the religious delusions entertained by the alleged lunatic, and said that Mr. Leach told him he believed he had offended the Almighty upon one occasion by cutting off his beard, and that he would rather go to the stake than do so a second time. This witness was also of opinion that Mr. Leach was of unsound mind.

Cross-examined.—The manner in which he obtained access to Mr. Leach was by representing that he and those who accompanied him were a deputation connected with some schools in the parish. He made this false representation on account of his being informed that he was in possession of fire-arms. While witness was talking to him his two attendants pounced on him, and he then said there was no occasion for any violence, and that if they were armed with legal authority he would accompany them quietly, and they released him, and he made no resistance. He had no pistols on his person, but he found two pistols up-stairs, both of which were unloaded. He had a powder-flask upon his person, and he gave it to them. Before he went away, a ring was taken out of the wardrobe and given to one of the maid-servants, and Mr. Leach told her to keep it, and he said he would write to her, but he never did so. The servant wrote one letter to him, and this letter was given to Mr. Leach's mother. In the course of the conversations he had with Mr. Leach, he said that he had told his sister that he intended to marry his servant, and that he was shut up immediately afterwards, and that this was the only cause of his being shut up.

Mr. Dewsnap, another surgeon, gave evidence of the same character as to the condition of mind of the alleged lunatic, and this closed the case on the part of the petitioner.

The rev. gentleman then submitted himself to examination, and he answered the questions put to him very readily, and made a long statement, no portion of which appeared to exhibit the slightest incoherence. He said he could not deny that, upon two occasions—namely, in 1841 and 1852—he had been very properly put under restraint, and he was much obliged to his friends for the course they adopted respecting him. As to the year 1853, when it was alleged he had another attack, he denied that he was ill at that time, and said that it was quite unjustifiable to place him under restraint; and the last time that he was placed in an asylum in 1857, he considered a most cruel and unjustifiable proceeding; and he said he considered it a monstrous proceeding that, upon the certificate of any two medical men, an Englishman could be seized and placed in a lunatic asylum, which he thought was quite as bad as the Inquisition. He was shut up for nearly a year in a box, and not allowed to communicate with anybody; and it was only at last, through the interference of the Commissioners of Lunacy, who he knew were satisfied that he was perfectly sane, that he was allowed to leave the asylum and go to a private lodging in the care of a keeper. He declared that the proceeding of making out that he was insane would never have been attempted if he had not expressed his determination to marry his servant-maid, and his family evidently thought it was a less evil that he should be incarcerated for life in a lunatic asylum, than that he should lose caste by marrying a person so much beneath his own condition. He then proceeded to declare that a great many of the notions he entertained and the expressions he had made use of had been very much misrepresented, and he denied ever having expressed an opinion that the millennium had arrived; and, on the contrary, he was satisfied that it had not, although he certainly did believe that it was approaching. He then proceeded to argue, very ingeniously, that there was no harm in having his servants to take meals with him, and he said he was first induced to do so in order to annoy his mother, who had insulted him, and he merely desired to show that he was determined to be master in his own house. He admitted that he really believed that he was converted by the appearance of our Saviour upon the cross to him twenty-seven years ago, and that since that period he had been under the peculiar influence of the Holy Spirit, and that every act of his life was performed under its dictation. He considered this was merely the result of his earnest prayers to the Almighty, and he believed that any other man might obtain the same gift who prayed with equal earnestness and sincerity, and he said he hoped that in the nineteenth century this would not be considered a proof of insanity.

Mr. Coleridge then summed up the case for the petitioner.

Mr. Montague Chambers made a most eloquent and powerful address to the jury on behalf of his client. He said that although counsel practising at the bar of England frequently had disagreeable and responsible duties cast upon them, and had to deal with questions involving an enormous amount of property, and also the forfeiture of life, he did not know of any more perplexing duty that could fall to the lot of an advocate than to endeavour to take off the erroneous impression as to the sanity of an individual who, by the strange law of England, was placed in the position of Mr. Leach. Every unfortunate person who was the subject of an inquiry of this kind, whether sane or insane, entered the room as a culprit—as a man who had been already convicted; and he put it to them whether, when they first saw him come before them, they did not all turn their eyes towards him and regard him with commiseration and pity, and that they started with the idea that they were dealing with an insane man, or, at least, with one who stood in a suspicious position, and who had been already condemned. The learned counsel who appeared in support of proceedings of this kind generally treated the matter very lightly, as though it was one of very little importance, and the jury was told that the unhappy subject of the inquiry had been already declared to be insane by competent persons, and all they had to do was to ratify the decision that had been before arrived at. He earnestly hoped they would not do any such thing in the present case, and that before they consigned a man, whom he represented as one perfectly sane, to a state of miserable restraint for the rest of his life, they would see that the evidence justified them in coming to the conclusion attended with such a fearful result. He called upon them to protect his highly gifted but unfortunate client from so dreadful a fate, and to treat him as they should wish to be treated themselves under similar circumstances, and to pause before they consigned him to a lunatic asylum for the rest of his life. He could not help expressing his opinion that an attempt had never before been made to establish a case of insanity upon such slight grounds as the present. The chief points relied upon appeared to be that Mr. Leach entertained opinions upon religious and other subjects that were considered by those around him to be extraordinary and erroneous; but could it for a moment be argued that this was a sufficient ground for saying that a man was insane? The study of the mental powers and the progress of thought and action were the most wonderful subjects of consideration—every day, every hour, fresh ideas entered the human mind; and when they entered into the consideration of these subjects, the greatest philosophers were baffled, and it not unfrequently happened that one thought the other a fool on account of some opinion he might have expressed. He did not deny that Mr. Leach entertained some erroneous opinions, and that his conduct upon some occasions had been eccentric; but he did not believe there was a man in creation who had not some eccentricities, follies, and even absurdities; and yet no one would think of sending such men to a madhouse. It appeared to him that his learned friends sought to confound these erroneous opinions with delusions; and all he could say was, that if they were to make the entertaining erroneous opinions a ground for saying that a man was insane, that they would have to shut up nine-tenths of the world, and that they would not be able to build a madhouse large enough to hold those who were considered mad because they entertained extravagant and unusual notions upon the subject of religion. Upon what ground was Mr. Leach to be shut up as a madman for the rest of his life? It was admitted that he lived quietly, happily, and with goodwill towards all mankind; and he contended that there was not the slightest danger to the community in his being at large. Mr. Leach was the grandson of a baronet, who left a large fortune, of which his mother had the life-interest, and the money at her death would be divided between him and the other members of the family. He was educated as a clergyman, and he performed his duties in the most exemplary manner until the year 1841; when owing to accidental causes—to some heavy duties being cast upon him—his mind gave way, and he had an attack of what was called acute mania. There was, however, no chronic disease of the brain, and he soon recovered, and he then devoted himself to religious study; and this led to a second attack in 1850, from which he also speedily recovered, and there was not the slightest evidence to show that there had ever been any organic disease of the brain. The learned counsel then referred to the supposed delusions entertained by Mr. Leach, and said, that as to his belief that he was impressed with the Holy Spirit, that many eminent and gifted men entertained similar opinions, and it would be idle to say that upon this ground Mr. Leach ought to be considered insane. A great deal had been made of the fact of Mr. Leach having purchased so many as five pistols; and he could not help expressing his surprise that his learned friends should have endeavoured to make this circumstance of so much importance, for it was really to be explained in the most simple and rational manner. The fact was, that about the time of the shocking murder of the Rev. Mr. Hollest, at Frimley, by burglars in the dead of the night, Mr. Leach, in common with a great portion of the public, felt a good deal alarmed at the circumstance, and being about to remove to the country, he purchased a revolver for his protection. This was lost, and he then purchased a pair of small pistols, and he was induced by the gunsmith to purchase a second pair on account of their great beauty, and their having been exhibited at the Great Exhibition. It was clear, therefore, that his only object in purchasing these pistols was to protect himself, and there was not a tittle of evidence that he had on any occasion used or threatened to use them offensively against any individual. The learned counsel next referred to the fact of Mr. Leach having, in June, 1856, been allured to execute a solemn deed, under which he and his brother-in-law, Mr. Sidden, received nearly 1200*l*. each; and he put it to the jury whether, if his family believed him to be a madman, they would have permitted him to execute such an instrument. He did not wish to say a word that could be considered offensive to the scientific gentlemen who had been examined in support of the commission, because he knew them to be men of honour and integrity, and to possess high scientific attainments; but at the same time there could be no doubt that they confirmed the statement of the poet, "all things looked yellow to the jaundiced eye," and that when a mad doctor was requested to look at a patient who was considered insane, he immediately put on his yellow spectacles, and observing him through their medium, very readily came to the conclusion that he was in that condition. He should be glad to know how any of the jury would like to undergo the sort of examination to which this gentleman had been subjected day after day, and for hours together, upon the most private and delicate matters; such questions as, "Well, when are you going to cut off your beard?" "When are you going to give up the girl?" being put to him, and whether they thought they would be able to come out of it as well as he did; for, after all these long examinations, only a few isolated expressions were picked out, upon which these gentlemen founded their belief that Mr. Leach was insane. Dr. Winslow, a gentleman of high honour and integrity, felt himself bound to admit that when he was first consulted by the mother of Mr. Leach, he said he did not consider there were sufficient grounds for restraining his liberty, and advised his friends to wait till he committed some overt act of insanity; and what was that overt act? Why, the determination he expressed to marry his servant-maid; and this aroused the pride of the family, and they determined to prevent the marriage taking place by every means in their power; and what course did they take to effect their object? Dr. Winslow, like an honourable man, declined to act, and advised that two independent medical men should be called in to sign the certificate of insanity; and who was one of those "independent" men? Why, Mr. Gray, the former assistant of Mr. Sidden, the brother-in-law of Mr. Leach, and whom they had not dared to put in the witness-box on the present occasion. Did the jury doubt for a moment that but for this intended marriage Mr. Leach never would have been interfered with, but would have been allowed to live as he had done before, and there never would have been any attempt to make out that he was insane? The learned counsel then referred to the question of the property to which Mr. Leach would become entitled on the death of his mother, and called the attention of the jury to the fact that if he should die unmarried and without a will, both which events were very likely to arise in the event of his being declared insane and consigned to a lunatic asylum, no less a sum than 30,000*l*. would go to his sister, and consequently come into the possession of her husband, Mr. Sidden, who had taken a most active part in these proceedings. The learned counsel concluded a very able speech by stating that he felt he was performing a most solemn duty on the present occasion, and he earnestly entreated the jury to weigh well all the evidence that would be laid before them before they returned a verdict the effect of which would be to place this unfortunate gentleman under the ban of being a lunatic for the rest of his life.

EVIDENCE AGAINST THE LUNACY.

Dr. Harrington Tuke, of Chiswick, was then examined. He stated that he was a physician, and son-in-law to Dr. Conolly, and had been a pupil of that gentleman. He had had the charge of an extensive lunatic asylum for eleven years, and had had a great deal of experience in the treatment of persons in that condition. He had interviews with Mr. Leach on the 10th, the 13th, and the 19th of May, for the purpose of ascertaining the state of his mind. When he first saw him he told him his object, and apologized for the questions that he said he should be compelled to put to him. He first alluded to his beard, and he said that many men wore their beards, and he saw no reason why he should not do so; but he did not consider the wearing of the beard as at all essential to salvation. On reference to his acting under the influence of the Holy Spirit in all his actions, he said that although he believed that he did so, still he considered that he was fallible like other men, and that he was equally liable to impulse, and, he added, that if the dictates of this Spirit were not rational or right, he certainly should not obey them. The witness said that he conversed with Mr. Leach upon the subject of the millennium, and he considered that his opinions upon that subject were quite correct, and even better than he could have expressed himself. He appeared to think that it might come in fifty or a hundred years, or on the morrow, but he expressed a decided opinion that it had not yet arrived. In reference to the purchase of the pistols, he said that his house had been broken into, and that he bought them for his protection; and upon one occasion he practised with them in his garden, but finding there was a public pathway near the spot, and that it might be dangerous to discharge pistols in such a place, he never did so again. Mr. Leach, in the course of the conversation, said that the marriage was the first thing, and the second thing, and the third thing, and that this was the only reason why his mother had shut him up, and he complained of her cruelty for doing so. Witness observed that it was an extraordinary act for a clergyman to marry one of his domestics, and he replied that ninety-nine men out of a hundred would think the same thing, but they did not know the circumstances. The fact was, he was isolated from the world, he never had the society of any ladies, and he really believed that this young woman would make him a good wife. He then asked Mr. Leach whether he did not desire to be reconciled to his mother, and he said he could not talk of reconciliation when the foot of his adversary was on his neck, and his sword was at his throat, and while a great struggle was going on, but when it was over he would gladly entertain the question of reconciliation. He said that he did not see any harm in an old man and a religious man kissing his servants, and that he never intended anything improper by doing so. He kissed the one he intended to marry upon the lips, and the other on the cheek; and on witness asking him if he intended to do the same after he was married, he replied that his wife would take care he did not do that (a laugh). In the course of the conversation he said that Mr. Leach went through the first proposition of Euclid from memory, working the letters and correct angles. Mr. Leach also talked about his property, and seemed to perfectly understand the value of money. The witness stated that in his opinion the first attack of mania in 1841 arose from excessive nervous excitement, and was not connected with any disease of the brain: and that the second, in 1852, was occasioned by over study, which frequently produced such a result; and he concluded by stating that, in his opinion, Mr. Leach at the present moment was of perfectly sound mind, and quite competent to manage and dispose of his property.

Upon being cross-examined, Dr. Tuke said that it was possible that a conversation might go on for a whole day with a lunatic without his insanity being discovered, unless his particular delusion was touched upon. He should consider it very extraordinary conduct in a clergyman to play at cards with his servants until a late hour of the night, and reading hymns during the deals very extraordinary conduct; but he should not, in the absence of explanation, come to the conclusion that a person who so acted must necessarily be insane. He had never seen a case where a lunatic was altogether able to conceal his delusions; but he did not observe such an attempt on the part of Mr. Leach. On the contrary, he appeared anxious to converse upon every subject that was suggested. The result of what he had seen of him was, that he considered he did not require the least supervision, and that he was as fit to be trusted with the possession of pistols, gunpowder, and bullets, as any other man, and he did not believe there was the least chance of his committing any act of violence either to himself or to others.

Dr. G. Johnson deposed that he was one of the physicians of King's College Hospital, and had had a good deal of experience in cases of insanity and acute mania. He had had three private interviews with Mr. Leach. The first was on the 8th of April, when he conversed with him upon the subjects that were supposed to form the ground for considering that he was insane. The result of the whole of his conversations with Mr. Leach was, that he was of opinion that he was of sound mind and perfectly competent to manage his own affairs, and he did not discover that he was labouring under any delusion. With regard to the vision in the pulpit, Mr. Leach only professed to have had a mental vision of our Saviour; and he said that he never intended it to be understood that he had seen our Saviour corporeally at the time in question. With regard to the millennium, Mr. Leach said that he did not believe that it had arrived, but that it was dawning, and would soon arrive. He also said that he never considered that he was obeying the Divine command in wearing his beard, although to a certain extent he felt that it was a point of conscience. He said that he had bought the pistols to protect his person and his property, and he did not utter a word that induced witness to believe that he was at all likely to commit an act of violence either upon himself or others; but, on the contrary, he seemed to be a remarkably quiet and inoffensive man. The witness then stated that he gave him exactly the same account with reference to the other supposed delusions that he did to Dr. Tuke; and he added that he did not appear to entertain any greater amount of animosity towards his mother than might reasonably be supposed would be entertained by a man who felt that he had been unjustly placed under restraint.

Cross-examined—Witness could not see anything in Mr. Leach that was different to other men. He went to see him on the first occasion with some suspicion in his mind, but if he had met him casually in a drawing-room he should not have thought there was anything extraordinary about him. Mr. Leach might have told him that he had the Divine sanction for wearing his beard, or words to that effect. He also seemed to think that it was more natural to wear the beard than not to do so.

Re-examined—In Southey's "History of Wesley and the Progress of Methodism," there were several instances of persons being converted by visions such as that described by Mr. Leach. All the impulses of Mr. Leach appeared to induce him to do good and never to do evil, and nothing that he had heard in the course of the present inquiry in any way tended to alter his original opinion. He considered Mr. Leach a very well informed, intelligent man. If he had met him in an ordinary drawing-room, he should have considered him an extraordinary man; but if he had met him in a drawing-room where there were none but learned and scientific men, he should have thought him quite at home, and neither his beard nor anything else about him would have excited the least suspicion in his mind respecting his sanity.

Mr. Fuller, a practitioner at St. John's Wood, gave similar evidence to that of Dr. Johnson. He said he had three long conversations with Mr Leach in January, and on the 14th and 18th of May, of the present year, and the result he arrived at was that he was perfectly sane, and he was quite astonished that such a man should be even under the surveillance of a keeper. The most that could be said was that Mr. Leach was an eccentric man; but he did not consider that he was labouring under a single delusion. He added that he believed Mr. Leach to be as harmless as a lamb, and that he was incapable of injuring any one.

Cross-examined—He put no question to Mr. Leach as to his opinion when a man forfeited his life, but he did say that he considered that if he were to shoot a keeper who came to take him, he should be guilty of murder, and should be executed. In the course of the conversation he had with Mr. Leach he said that he played at cards with his servants as a relaxation after severe study. Witness told him that he still thought it was inconsistent with his position, and he replied that no one could come to the correct opinion as to his feelings, and the position in which he had been placed for a great many years without any society, and having no one about him but his servants, and that when his mother left him he was still more lonely. If Mr. Leach had said that he played cards with his servants partly from his knowledge of the Scriptures, he should consider it, without explanation, inconsistent with the idea that he was of sound mind; but with Mr. Leach's peculiar religious opinions, he should consider it necessary to ask him to explain what he meant.

Re-examined—In witness's opinion it was a cruelty to lock up such a man as Mr. Leach. His attention was first called to the case by Mr. Clerk, Mr. Leach's bootmaker, and it was in consequence of his humane interference that he went to see him upon the first occasion. In his opinion Mr. Leach was a man of great piety, sound judgment, and great learning, and it was a pleasure to converse with him.

Dr. Seymour, formerly one of the Commissioners of Lunacy in the metropolis, deposed that he had for eight years acted in that capacity, and he had also had a great deal of experience in other respects in connexion with lunatics. He was first instructed to see Mr. Leach on the part of his mother, through the agency of Dr. Winslow. The report he made was that his irregularities were not, at the time he saw him, of such a character as would induce any jury to find that he was insane. He stated that his views of religion were the same that were entertained by many other persons; and as to his marrying his servant, although it was a very foolish thing, and contrary to their received notions of propriety, still it did not at all establish his insanity; and if such a proceeding were allowed to make out that a man was insane, all he could say was that half of Westminster Hall would be in confinement. (A laugh.) On the second occasion when he saw him, he thought he was much better; his conversation was pleasing; he was a very humane man, and he saw no reason whatever for placing him in confinement. The last time he saw Mr. Leach was in December last, and after he had made his report, the solicitors for Mrs. Leach did not communicate with him, and he was not aware of the former trial having taken place, and he had been suddenly summoned this morning to give evidence. Mr. Leach's religious opinions were certainly peculiar, but not at all more so than were entertained by many persons with whom he was acquainted, and none of them were of a character that was likely to induce him to commit any act of violence. He was quite aware of the nature of the supposed delusions entertained by Mr. Leach, and he touched upon the whole of them, and the opinion he arrived at was, that no twelve men would find him of unsound mind and incapable of managing his affairs.

In cross-examination Dr. Seymour admitted that he had written a letter, March, 1857, in which he stated that Mr. Leach was of unsound mind, and in capable of managing his affairs; but he also said that he did not think any jury would find he was in that state. When he saw Mr. Leach the second time, in December, according to his opinion he was better. It was his unbiassed and clear opinion, in March, 1857, that Mr. Leach was of unsound mind and incapable of managing his own affairs, and that his family acted wisely and humanely in the course they had taken. Witness considered that Mr. Leach's religious opinions were very much modified when he saw him at Christmas last. In March he appeared to consider that the millennium had arrived or was imminent, but he did not appear to think this was the case in December, and he was much more gentle. At first he appeared to consider that he wore his beard under the authority of the Bible, but the second time he said that he wore his beard because other people wore theirs, and he did not see why he should be looked upon as insane on that account. The witness further stated that a singular feature in Mr. Leach's character was his extreme harmlessness.

Re-examined—He was instructed, in the first instance, that Mr. Leach had struck his mother, but it turned out that there was no foundation whatever for this statement. There were many people moving about the world and managing all their affairs, who, to his knowledge, entertained quite as extraordinary opinions on the subject of religion as Mr. Leach did, and he should be very sorry to say that these persons were insane on that account. Taken as a whole, he thought at first that Mr. Leach had very peculiar opinions, and, coupled with the knowledge of the two previous attacks, he was of opinion that Mr. Leach was of unsound mind in March, 1857, and that he had better remain quietly in the asylum for a short time. After the second visit he considered that there was no danger in his being at large, and that he was quite competent to manage his affairs; and if he were confined in an asylum over which witness had control, he should have no hesitation in setting him at liberty.

By the Commissioner—He had a very long professional interview with Mr. Leach in March, and he then came to the conclusion that he was insane, but he should have felt a difficulty in signing a certificate for his removal to a lunatic asylum, and he should rather have had him placed under the charge of his friends or in the care of an attendant.

Mr. Cotton, a tailor, who had occasionally worked for Mr. Leach for sixteen years, proved that he ordered what he required and paid him regular, and appeared quite competent to manage his affairs. He said that he first heard Mr. Leach was taken to a lunatic asylum between Christmas and March, 1857. He was a very kind and benevolent man, and he considered that he would not injure any one.

Mr. Carey, another tailor, proved that he was in the habit of communicating with Mr. Leach up to the time when he was taken to the lunatic asylum, and he and every one else in the neighbourhood were quite astonished that such a course should have been taken respecting him. He had some clothes to make for him at the time, and he went to Dr. Winslow's asylum about three weeks after he had been there, and saw him upon the subject of these clothes. He was told that he would not be allowed to see Mr. Leach again unless he brought a letter from his mother, and as he knew it was no use asking for that, he never saw him again.

Mr. Smith, a builder at Hammersmith, gave similar evidence, and he also proved that there were a great many robberies at that place, and that a good many gentlemen living there purchased fire-arms. Mr. Leach was very correct in all his dealings, and he considered that he was perfectly sane.

Mr. Clerk, a bootmaker, deposed to the same effect, and also stated that he was so satisfied that Mr. Leach was incapable of committing any act of violence, that he should have no objection to sleep in the same room with him if he had loaded pistols in his possession.

Mr. J. U. Romer, a gentleman who lived in an adjoining villa, proved that his house was attempted to be robbed, and that Mr. Leach's was also broken into. He saw Mr. Leach about every day up to the time of his being taken away, and he always considered him a very sensible, sane man, and quite competent to undertake the management of his affairs. His mother lived with him a great portion of the time, and she never made any complaint of the condition of his mind, or of any necessity that existed for his being placed under control.

Dr. Conolly, the consulting physician at Hanwell for twenty years, was then examined, and he stated that in his opinion Mr. Leach was quite competent to manage his affairs. He said that he had very long conversations with him, and he allowed him to go on talking for a long time, and he never exhibited the least incoherence. He did not appear to desire to conceal anything, but candidly admitted that upon two occasions he had been properly placed under restraint.

Cross-examined—He had advised at one time that Mr. Leach should be under surveillance, or that he should travel accompanied by a medical gentleman; but his opinion now was that any surveillance would be irksome to him, and that it was quite unnecessary.

Re-examined—He distinctly stated on the former occasions, that in his opinion the best thing that could be done was to release Mr. Leach from restraint.

By the jury—If there had been any trace of insanity in the mind of Mr. Leach, the severe test that had been applied to him of confining him in a lunatic asylum for so long a period was most calculated to bring it out.

The following letter, addressed by Mr. Leach to his brother-in-law, Mr. Sidden, while in confinement at Dr. Winslow's, was then put in and read:—

"Why, Sidden, what a man you are to act as you have done towards me! I am really annoyed at you, and hardly know how to write from impatience and disgust. To cause me to be put under restraint, taking such a cowardly advantage of my isolated position—a marvellous thing, indeed, that you should have such power in this free country. But for my projected marriage, such a proceeding could never have entered your head. Why, it was only on the Saturday previously that I had the long conversation with Laura, when I waited for you till half-past five, that we might talk the matter over. What was there in my manner or language that could indicate the shadow of a taint of insanity? I can confidently assert—nothing; and so would Laura. But it seems she thinks it less disgrace to live with a woman without marriage, or to visit houses where men are in the habit of gratifying their passions, than to contract a lawful marriage with a person of different station from myself. I hold an opposite opinion, and for that I am deprived of my liberty—a most cruel and wicked step on your part, for which you will one day have to answer to your shame. There is a Power above who will sooner or later right the oppressed, and in that Righteous Power I put my implicit trust. This house is not intended to hold persons like myself, who happen to give offence to their mother and sisters, but for those unfortunate ones who, under delusion, think their right hand their left, or fancy themselves emperors or kings, &c. Nobody ever thought of imprisoning Charles Mathews when he married Madame Vestris, or Alfred Montague when he united himself to one of the same sort of women. And though there is a religious motive associated with mine, if that would indicate insanity, you might as well imprison the whole body of Quakers, of Methodists, of Swedenborgians, of Mormonites, and I know not how many more, who act according to their consciences, and believe that they act more or less under Divine influence in their every-day proceedings. Think of the Quakers, who believe in what they call the inward spirit, and never speak at their public meetings unless they are, or think they are, moved by the Holy Spirit. Women are allowed thus to speak as well as men. How ridiculed they are, and have been, and persecuted, everybody knows; but we have lived to see the day when they and all religious sects are freely tolerated. It is grievous to think that for my temper on the Friday previous, and my divulging my intended marriage—which, by the way, if I had chosen I could have contracted secretly—neither you nor any one else would have cared more than you have ever cared for my religious opinions or conduct; that is to say, not a twopenny piece. I do hope and pray that both Winslow and Bartlett will be very soon convinced of my perfect sanity, and that, as Englishmen and Christians, they will hasten to wash their hands of this matter. If not, and it were possible you could succeed in keeping me here, why, then, I might as well be in Rome, and imprisoned in the Inquisition for denying the supremacy of the Pope or transubstantiation, save and except that there is no rack or torture, but, on the contrary, much comfort, and every one trying to do the best they can for us. Sidden, cease, I beseech you, from this unmanly conduct, and show yourself a man, and somewhat worthy to live in such a country as this, where Sabbaths, and clergy, and Bibles compel almost every man to know right from wrong; where responsibility is therefore greater, and retribution in cases of injury must be most terrible."

Mr. Garth then proceeded to sum up the case of the alleged lunatic, and he urged that it had been most clearly established that there was not the slightest pretence for saying that he was of unsound mind, and that he was entitled at once to be set at liberty.

Mr. Bovill then made a most able reply upon the whole case. He particularly remarked upon the servants who were with Mr. Leach from the period of his mother leaving the house until he was removed to Dr. Winslow's asylum not having been called as witnesses to show what his conduct really was during that period; and he said that the only inference that could be drawn from their absence was, that if they had been called they would have been compelled to admit that he was in a state of insanity. The learned counsel then commented upon the evidence that had been adduced on both sides, and concluded by expressing his opinion that there could be no reasonable doubt of the insanity of this unfortunate gentleman, and that the jury would have no alternative but to return a verdict to that effect.

The Commissioner addressed a very few observations to the jury, and at half-past seven o'clock the room was cleared, and they proceeded to deliberate upon their verdict.

In a very few minutes the court was re-opened, when the foreman announced that, by a majority of nineteen to four, they were of opinion that Mr. Leach was of sound mind, and perfectly competent to manage his affairs.

There was a burst of applause when the verdict was pronounced.

